# Neutrophils as key regulators of tumor microenvironment in breast cancer: a focus on N1 and N2 polarization

**DOI:** 10.1097/MS9.0000000000003269

**Published:** 2025-04-10

**Authors:** Emmanuel Ifeanyi Obeagu, Christian C Ezeala

**Affiliations:** a Department of Biomedical Laboratory Science, Africa University, Mutare, Zimbabwe

**Keywords:** breast cancer, N1 polarization, N2 polarization, neutrophils, tumor microenvironment

## Abstract

Neutrophils, the most abundant type of white blood cells in the human body, play a vital role in the immune response against infections and tissue injury. However, in the context of cancer, their function becomes more complex and context-dependent. In breast cancer, neutrophils are key players in shaping the tumor microenvironment (TME), a highly dynamic ecosystem where various cell types, extracellular matrix components, and soluble factors interact to influence tumor progression, immune evasion, and metastasis. Neutrophils in the TME are not just passive participants but actively engage in altering tumor biology, either supporting or inhibiting tumor growth depending on their polarization status. Neutrophils exhibit plasticity in their phenotype and function, which can be categorized into two polarized forms: N1 and N2. N1 neutrophils are associated with antitumor responses, promoting immune activation, direct cytotoxicity against tumor cells, and facilitating the clearance of cancerous cells through the release of reactive oxygen species, cytokines, and chemokines. Conversely, N2 neutrophils contribute to tumor progression by fostering an immunosuppressive environment, promoting angiogenesis, enhancing tumor cell migration and invasion, and aiding in the establishment of metastatic niches. This dichotomy of neutrophil polarization plays a crucial role in determining breast cancer progression, metastasis, and response to treatment.

## Introduction

Neutrophils, the most abundant type of white blood cells in the human body, play a vital role in the immune response against infections and tissue injury. However, in the context of cancer, their function becomes more complex and context-dependent. In breast cancer, neutrophils are key players in shaping the tumor microenvironment (TME), a highly dynamic ecosystem where various cell types, extracellular matrix (ECM) components, and soluble factors interact to influence tumor progression, immune evasion, and metastasis. Neutrophils in the TME are not just passive participants but actively engage in altering tumor biology, either supporting or inhibiting tumor growth depending on their polarization status^[1-3]^. Neutrophils exhibit plasticity in their phenotype and function, which can be categorized into two polarized forms: N1 and N2. N1 neutrophils are associated with antitumor responses, promoting immune activation, direct cytotoxicity against tumor cells, and facilitating the clearance of cancerous cells through the release of reactive oxygen species (ROS), cytokines, and chemokines. Conversely, N2 neutrophils contribute to tumor progression by fostering an immunosuppressive environment, promoting angiogenesis, enhancing tumor cell migration and invasion, and aiding in the establishment of metastatic niches. This dichotomy of neutrophil polarization plays a crucial role in determining breast cancer progression, metastasis, and response to treatment^[4,5]^. The polarization of neutrophils is influenced by various factors within the TME, including cytokines, growth factors, and tumor-derived signals. Tumor cells can secrete molecules that skew neutrophils toward the N2 phenotype, thereby fostering an immunosuppressive microenvironment conducive to cancer progression. On the other hand, the immune system can also induce polarization toward N1 neutrophils, supporting antitumor immunity^[6]^.

**HIGHLIGHTS**

- N1 vs. N2 Polarization: N1 neutrophils exhibit anti-tumor activity, while N2 promote tumor growth and immune evasion.
- TME Dynamics: Tumor microenvironment (TME) influences N1/N2 polarization.
- Immunosuppression: N2 neutrophils suppress T-cell responses.
- Cytokine Regulation: IL-8, TGF-β, and GM-CSF drive N2 polarization.
- Therapeutic Targeting: Modulating neutrophil polarization offers novel breast cancer treatment strategies.

Research has demonstrated that the balance between N1 and N2 neutrophils significantly impacts breast cancer progression and metastasis. A predominance of N2 neutrophils in the TME has been associated with poor prognosis, as these cells contribute to immune suppression, tumor cell escape from immune surveillance, and the promotion of an invasive phenotype. In contrast, the presence of N1 neutrophils has been linked to better clinical outcomes, as these cells are involved in targeting and eliminating tumor cells through immune-mediated mechanisms. Consequently, neutrophil polarization could serve as a potential biomarker for predicting breast cancer prognosis and response to therapy^[7,8]^. Breast cancer therapies, including chemotherapy, immunotherapy, and targeted treatments, often fail to produce lasting results due to the complex interactions between the tumor and the immune system. One critical challenge is the immunosuppressive environment created by N2 neutrophils, which limits the effectiveness of these treatments. Reprogramming neutrophils toward the N1 phenotype could enhance the antitumor immune response, improve treatment efficacy, and overcome resistance mechanisms. Therefore, strategies aimed at modulating neutrophil polarization are emerging as a promising avenue for improving breast cancer therapies^[9,10]^.

## Aim

The aim of this review is to explore the role of neutrophils, specifically N1 and N2 polarization, in the TME of breast cancer and their impact on tumor progression, metastasis, and treatment outcomes.

## Rationale

The rationale for this review stems from the increasing recognition of neutrophils as key regulators of the TME in breast cancer. Neutrophils, traditionally known for their role in innate immunity, exhibit a high degree of plasticity in the TME, where they can polarize into two distinct phenotypes: N1 and N2. N1 neutrophils are considered pro-inflammatory and antitumor, while N2 neutrophils contribute to immune suppression, tumor progression, and metastasis. Recent advances in cancer immunology have highlighted the potential of modulating neutrophil function to improve therapeutic outcomes. However, the mechanisms behind neutrophil polarization in the TME remain complex, and therapeutic interventions aimed at manipulating this balance are still in their infancy. By focusing on the polarization of neutrophils, particularly the modulation of N1 and N2 phenotypes, this review seeks to fill gaps in current knowledge and identify strategies that could enhance antitumor immunity, overcome tumor resistance, and complement existing breast cancer therapies. In addition, neutrophils are highly abundant and relatively easy to target compared to other immune cell populations, making them an attractive candidate for therapeutic intervention. Therefore, this review aims to provide a comprehensive understanding of neutrophil polarization in breast cancer, the potential of targeting these cells to reshape the TME, and the challenges that need to be addressed to fully exploit their therapeutic potential. By synthesizing current findings, this review hopes to guide future research efforts in developing effective treatments that can harness neutrophils as powerful allies in the fight against breast cancer.^[11-15]^

## Review methodology

This review was conducted by systematically gathering, evaluating, and synthesizing existing literature on neutrophil polarization, specifically focusing on N1 and N2 phenotypes, in the context of breast cancer. The methodology was designed to provide a comprehensive overview of current knowledge on the role of neutrophils in breast cancer progression, metastasis, and treatment outcomes, as well as to identify potential therapeutic strategies targeting neutrophil polarization.

## Literature search

A detailed search of peer-reviewed articles, reviews, clinical studies, and preprints was conducted using major scientific databases, including PubMed, Scopus, and Google Scholar. The search was limited to articles published in the last 10 years to ensure the inclusion of the most recent findings. Keywords used in the search included “N1 neutrophils,” “N2 neutrophils,” “tumor microenvironment,” “breast cancer,” “neutrophil polarization,” “cancer progression,” “metastasis,” and “immunotherapy.” Studies were selected based on their relevance to the topic, with an emphasis on understanding the polarization of neutrophils in breast cancer and its impact on tumor progression and therapy response.

## Inclusion and exclusion criteria

Studies were included if they focused on the role of neutrophils in breast cancer, particularly in terms of their polarization into N1 and N2 phenotypes, their impact on the TME, and their effect on cancer progression, metastasis, and therapy. Articles exploring therapeutic strategies that target neutrophil polarization or immune modulation in cancer were also included. Exclusion criteria included studies not related to breast cancer, those focusing on neutrophil biology in non-cancer contexts, and articles lacking detailed information on neutrophil polarization or its specific role in breast cancer.

## Neutrophil polarization in the TME

Neutrophils, as the first responders to infection and injury, have long been recognized for their role in innate immunity. However, within the context of cancer, their function is far more nuanced, particularly in the TME, where they exhibit remarkable plasticity in their phenotype and function. Neutrophil polarization refers to the process by which neutrophils differentiate into distinct functional subtypes, each contributing differently to the tumor progression. In the TME, neutrophils can be polarized into two main states: the antitumorigenic N1 phenotype and the protumorigenic N2 phenotype. This polarization process is driven by various cues from the tumor and surrounding stromal cells, including cytokines, growth factors, and ECM components, which ultimately dictate whether neutrophils will support tumor immunity or promote tumor progression^[16-18]^. The N1 phenotype is often associated with the activation of antitumor immune responses. N1 neutrophils are typically characterized by their ability to produce high levels of ROS, pro-inflammatory cytokines, and chemokines that help recruit other immune cells, such as T cells and natural killer (NK) cells, to the tumor site. Additionally, N1 neutrophils can directly kill tumor cells through the release of cytotoxic molecules like granules containing enzymes (e.g., myeloperoxidase) and proteases, as well as through the formation of neutrophil extracellular traps (NETs). These immune responses work together to inhibit tumor growth and metastasis, making N1 neutrophils essential players in immune surveillance and tumor suppression^[19,20]^.

Conversely, the N2 phenotype is typically associated with a pro-tumorigenic role, facilitating tumor progression and metastasis. N2 neutrophils exhibit an immunosuppressive profile, secreting cytokines like interleukin-10 (IL-10) and transforming growth factor-beta (TGF-β), which promote immune tolerance and suppress the function of cytotoxic T cells and NK cells. They also support tumor angiogenesis by secreting vascular endothelial growth factor (VEGF) and other pro-angiogenic factors, which enable tumor vascularization and facilitate metastasis. Additionally, N2 neutrophils contribute to tissue remodeling and ECM degradation, processes that promote the invasion and migration of tumor cells. The presence of a high proportion of N2 neutrophils in the TME is often linked to poor prognosis, as these cells foster an environment conducive to immune evasion, tumor cell survival, and metastatic spread^[21-23]^. The polarization of neutrophils is not an all-or-nothing process; instead, it exists along a spectrum, with cells displaying intermediate phenotypes. In the TME, factors such as hypoxia, the presence of inflammatory cytokines (e.g., interleukin-6 (IL-6), tumor necrosis factor-alpha (TNF-α)), and other signals like granulocyte colony-stimulating factor (G-CSF) can dictate the polarization of neutrophils toward either N1 or N2 phenotypes. Tumor cells can actively manipulate neutrophil polarization by secreting cytokines and chemokines that skew neutrophils towards an N2-like, tumor-promoting phenotype. This flexibility in neutrophil polarization suggests that the TME is a highly dynamic environment, where neutrophils can rapidly adapt to different microenvironments and switch between functional states depending on the cues they receive. Understanding the regulatory mechanisms of neutrophil polarization in the TME is crucial for designing targeted therapies that could potentially shift the balance from N2 to N1 neutrophils, thus enhancing the antitumor immune response^[24-26]^. In addition to tumor-derived signals, other factors, such as the presence of microbial infections or the composition of the gut microbiota, can also influence neutrophil polarization. For instance, certain bacterial infections in the TME can activate neutrophils and prime them to adopt an N1 phenotype, contributing to the elimination of tumor cells. The complex interplay between the immune system, the tumor, and the microbiome underscores the intricate regulatory network that governs neutrophil polarization. As such, understanding these external and internal cues, and how they influence neutrophil function in the TME, represents a promising area of investigation for developing immunotherapies aimed at modulating neutrophil activity in cancer^[27,28]^.

## Role of N1 neutrophils in breast cancer

N1 neutrophils, often referred to as the “antitumor” phenotype, play a critical role in the immune response against breast cancer by exerting cytotoxic effects on tumor cells and promoting antitumor immunity. These cells are considered to be highly active in fighting cancer, as they are capable of triggering several immune responses that can limit tumor growth and metastasis. N1 neutrophils are typically characterized by their ability to produce high levels of ROS, pro-inflammatory cytokines, and chemokines, all of which contribute to an immune microenvironment that is hostile to tumor progression^[29,30]^. One of the key functions of N1 neutrophils in breast cancer is the direct killing of tumor cells. N1 neutrophils are equipped with cytotoxic granules containing enzymes such as myeloperoxidase and elastase, which can be released to target and destroy cancer cells. Additionally, these neutrophils are capable of forming NETs, which are networks of extracellular fibers made of DNA and proteins that entrap and kill pathogens. In the case of cancer, NETs can also entrap and directly target tumor cells, limiting their ability to proliferate and invade surrounding tissues. This NET-mediated response serves as a critical barrier to cancer cell dissemination and metastasis^[31,32]^. Furthermore, N1 neutrophils contribute to the recruitment and activation of other immune cells, including T cells and NK cells, which are pivotal in the body’s defense against cancer. By secreting cytokines such as TNF-α, interleukin-12 (IL-12), and interferon-gamma (IFN-γ), N1 neutrophils facilitate the polarization and activation of cytotoxic T cells and NK cells, creating a pro-inflammatory microenvironment that promotes tumor immune surveillance. Additionally, these cytokines contribute to the enhancement of antigen presentation by dendritic cells and macrophages, further amplifying the adaptive immune response and ensuring a robust and effective attack on the tumor^[33,34]^.

The presence of N1 neutrophils in the TME is generally associated with favorable outcomes in breast cancer. High numbers of N1 neutrophils correlate with improved prognosis, as they support an immune environment that is less conducive to tumor survival and growth. N1 neutrophils can also limit tumor angiogenesis by secreting factors that inhibit the formation of new blood vessels. As tumors often rely on angiogenesis for nutrient and oxygen supply, the inhibition of this process by N1 neutrophils can restrict tumor growth and limit metastatic potential^[8,35]^. However, the antitumor effects of N1 neutrophils are highly dependent on the specific cytokine milieu within the TME. Factors such as IL-6, G-CSF, and TGF-β can influence neutrophil behavior and polarization. For example, chronic inflammation and the presence of certain cytokines can skew neutrophils towards a more immunosuppressive N2 phenotype, which diminishes their antitumor activity. Additionally, while N1 neutrophils have potent tumoricidal capabilities, their efficacy can be hindered by the immunosuppressive nature of the TME, including the presence of regulatory T cells (Tregs) and myeloid-derived suppressor cells, which limit their ability to function optimally^[36,37]^.

## Role of N2 neutrophils in breast cancer

N2 neutrophils, often referred to as the “pro-tumor” phenotype, play a crucial role in promoting breast cancer progression, metastasis, and immune evasion. While N1 neutrophils exhibit antitumor activity, N2 neutrophils are generally associated with immune suppression and tumor growth. These cells are often found in higher numbers in tumors and are recruited by tumor-derived signals, contributing to a tumor-supportive environment that aids in cancer cell survival, proliferation, and dissemination^[38]^. One of the primary roles of N2 neutrophils in breast cancer is to promote tumor angiogenesis. By secreting factors such as VEGF, N2 neutrophils enhance the formation of new blood vessels that supply nutrients and oxygen to the growing tumor. This angiogenic activity not only supports tumor growth but also provides a route for cancer cells to spread to distant organs. In addition to promoting angiogenesis, N2 neutrophils contribute to tumor progression by secreting matrix metalloproteinases (MMPs), which degrade the ECM and facilitate tumor invasion and metastasis^[39]^.

N2 neutrophils also contribute to the suppression of the immune response within the TME. They secrete a range of immunosuppressive cytokines, including IL-10, TGF-β, and prostaglandin E2 (PGE2), which inhibit the activation of cytotoxic T cells and NK cells. Additionally, N2 neutrophils can recruit and activate regulatory T cells (Tregs), which further dampen the antitumor immune response. By creating an immunosuppressive microenvironment, N2 neutrophils not only protect the tumor from immune surveillance but also contribute to the development of resistance to therapies such as chemotherapy and immunotherapy^[14,40,41]^. Furthermore, N2 neutrophils are involved in the regulation of inflammation in the TME. While inflammation is a natural response to tissue damage or infection, chronic inflammation within the TME can be detrimental, as it promotes tumor progression, immune suppression, and metastasis. N2 neutrophils secrete pro-inflammatory cytokines, such as IL-1β and TNF-α, which can exacerbate the inflammatory milieu and create conditions that favor tumor cell survival and dissemination. This chronic inflammation not only supports the tumor’s growth but also creates a favorable niche for metastatic spread, especially to distant organs such as the lungs and liver^[26,42]^. N2 neutrophils also play a role in the establishment of a premetastatic niche, which is essential for the successful colonization of distant organs by cancer cells. By releasing factors that remodel the ECM and promote tissue remodeling, N2 neutrophils prepare distant organs for the arrival of circulating tumor cells (CTCs). These neutrophils enhance the ability of breast cancer cells to survive in the bloodstream and establish secondary tumors by secreting chemokines such as chemokine ligand 2 (CCL2), which recruit additional immune cells that support metastatic colonization^[34,43]^. Despite their pro-tumor properties, N2 neutrophils are not inherently malignant. Instead, their polarization and behavior are influenced by the TME. Factors such as cytokines, growth factors, and the presence of other immune cells in the TME drive the polarization of neutrophils toward the N2 phenotype. Tumors often exploit these signals to induce neutrophil polarization into a phenotype that favors tumor progression and immune suppression. This dynamic suggests that therapeutic interventions aimed at modulating neutrophil polarization could potentially alter the course of breast cancer progression^[27,44]^.

## Impact of neutrophil polarization on breast cancer metastasis

Neutrophil polarization plays a significant role in the progression and metastasis of breast cancer, influencing the TME in ways that can either promote or inhibit tumor spread. The polarization of neutrophils into either an N1 (antitumor) or N2 (protumor) phenotype can dictate the outcome of breast cancer metastasis. While N1 neutrophils typically have tumor-suppressive effects, N2 neutrophils contribute to a more supportive environment for cancer cells to invade surrounding tissues, spread to distant organs, and evade immune surveillance. The balance between these two phenotypes within the TME significantly affects the metastatic potential of breast cancer^[45,46]^. N1 neutrophils, known for their antitumor properties, play an essential role in preventing cancer metastasis by directly attacking and killing tumor cells. These neutrophils are equipped with cytotoxic capabilities, including the production of ROS and the formation of NETs, which can physically trap and eliminate tumor cells. Moreover, N1 neutrophils help recruit other immune cells, such as cytotoxic T lymphocytes (CTLs) and NK cells, which further contribute to tumor cell destruction. In addition to their direct cytotoxic functions, N1 neutrophils can also inhibit the process of angiogenesis, thereby restricting the growth of new blood vessels that tumors need for nutrients and oxygen. By limiting tumor vascularization, N1 neutrophils effectively reduce the ability of the tumor to spread^[47,48]^. In contrast, N2 neutrophils, which are often found in the TME of advanced and metastatic breast cancer, facilitate metastasis by promoting a variety of tumor-supportive processes. One of the key roles of N2 neutrophils in metastasis is their ability to enhance tumor angiogenesis. By secreting VEGF and other pro-angiogenic factors, N2 neutrophils promote the formation of new blood vessels that allow the tumor to expand and provide a route for CTCs to disseminate. N2 neutrophils also secrete MMPs, which degrade the ECM, allowing cancer cells to invade surrounding tissues and spread to other parts of the body. This degradation of the ECM is a critical step in cancer metastasis, as it enables the tumor cells to breach the basal membrane and enter the bloodstream or lymphatic system^[49,50]^.

**Figure 1.**
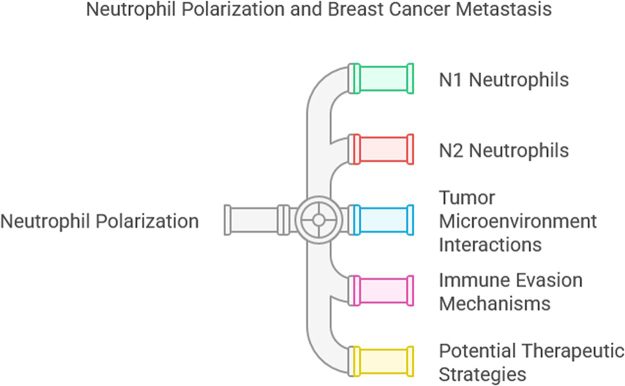
Neutrophils polarization and breast cancer metastasis

In addition to promoting angiogenesis and invasion, N2 neutrophils contribute to immune evasion, a crucial aspect of metastasis. By secreting immunosuppressive cytokines such as IL-10, TGF-β, and PGE2, N2 neutrophils inhibit the activation of cytotoxic T cells and NK cells, which are essential for eliminating metastatic tumor cells. Furthermore, N2 neutrophils can recruit regulatory T cells (Tregs), a subset of immune cells that suppress the immune response and protect the tumor from immune-mediated destruction. By creating an immunosuppressive environment, N2 neutrophils help tumor cells evade detection and destruction by the immune system, facilitating metastatic spread and reducing the effectiveness of therapies such as immunotherapy^[21,51]^. The impact of neutrophil polarization on breast cancer metastasis is not only determined by the quantity of N1 and N2 neutrophils within the TME but also by the interactions between these cells and other components of the immune and stromal microenvironment. Tumors can exploit various signaling pathways, including those mediated by cytokines, chemokines, and growth factors, to skew neutrophil polarization toward the N2 phenotype, thus fostering a pro-metastatic environment. This dynamic shift in neutrophil function reflects the plasticity of the immune response and its ability to adapt to the needs of the tumor. In this regard, understanding the signals that drive neutrophil polarization and the mechanisms by which these cells influence metastasis is crucial for the development of novel therapeutic strategies aimed at modulating neutrophil behavior^[33,52]^. (Fig. 1) shows Neutrophils Polarization and Breast Cancer Metastasis (provided by the authors).

## Emerging therapeutic targets in breast cancer

Breast cancer remains one of the most prevalent malignancies worldwide, with a highly heterogeneous nature that complicates treatment efforts. While conventional therapies, including chemotherapy, radiation, and hormone therapy, have significantly improved patient survival, resistance and recurrence remain major challenges. The TME plays a crucial role in driving breast cancer progression, and recent advancements in cancer research have highlighted the importance of identifying new therapeutic targets. Novel approaches aimed at disrupting key molecular pathways and cellular interactions within the TME are increasingly being explored as potential breakthroughs in breast cancer therapy.

## Targeting tumor-associated neutrophils (TANs) and the N1/N2 axis

Neutrophils, particularly TANs, are emerging as critical players in breast cancer progression. Their polarization into anti-tumorigenic (N1) and pro-tumorigenic (N2) subsets offers new avenues for targeted therapy. The shift toward the N2 phenotype is largely driven by tumor-derived factors, including TGF-β, which suppresses N1 neutrophil functions while enhancing the immunosuppressive and pro-metastatic properties of N2 neutrophils. Recent studies suggest that inhibiting TGF-β signaling could restore the anti-tumorigenic functions of neutrophils, reducing their contribution to tumor growth and metastasis. Small-molecule inhibitors such as galunisertib, a TGF-β receptor kinase inhibitor, have demonstrated promising preclinical results in reversing the N2 phenotype and promoting anti-tumor immunity. Additionally, the use of CXCR2 inhibitors to block neutrophil recruitment to the tumor site is another promising strategy to reduce the impact of pro-tumorigenic TANs in breast cancer^[8,53]^.

## Targeting tumor-associated macrophages (TAMs) and the CSF-1/CCL2 axis

Macrophages, particularly TAMs, are another key component of the TME that contribute to breast cancer progression. TAMs exist in two major phenotypes: M1 (anti-tumorigenic) and M2 (pro-tumorigenic). Similar to neutrophils, M2-polarized TAMs promote tumor growth, angiogenesis, and immune evasion. The CSF-1 and C-C motif CCL2 pathways have been identified as critical regulators of TAM recruitment and polarization. CSF-1R inhibitors, such as pexidartinib, have shown potential in depleting TAMs within the TME, thereby enhancing the efficacy of immunotherapies. Similarly, blocking CCL2 signaling with monoclonal antibodies has been explored as a strategy to disrupt the recruitment of pro-tumorigenic macrophages^[54-56]^.

## Targeting cancer-associated fibroblasts (CAFs) and the fibroblast activation protein (FAP)

CAFs play a crucial role in breast cancer progression by remodeling the ECM, promoting angiogenesis, and facilitating immune evasion. FAP, a key marker of CAFs, has been identified as a potential therapeutic target due to its role in tumor-stroma interactions. Targeting FAP with monoclonal antibodies, small-molecule inhibitors, and FAP-targeted chimeric antigen receptor T cells has shown promising results in preclinical models. FAP inhibitors, such as talabostat, have demonstrated the ability to disrupt CAF-mediated tumor support, reducing tumor growth and enhancing immune infiltration into the TME^[57,58]^.

## Exploiting the role of hypoxia and the hypoxia-inducible factor-1 alpha (HIF-1α) pathway

Hypoxia is a hallmark of the breast cancer microenvironment and plays a crucial role in tumor progression, metastasis, and therapy resistance. HIF-1α is a key transcription factor that regulates cellular responses to low oxygen levels, promoting angiogenesis, metabolic reprogramming, and immune suppression. Inhibiting HIF-1α has been explored as a strategy to sensitize tumors to existing therapies and prevent metastatic dissemination. Small-molecule inhibitors such as PX-478 and acriflavine have demonstrated potential in reducing HIF-1α activity, thereby impairing tumor adaptation to hypoxic conditions. Additionally, targeting metabolic pathways influenced by hypoxia, such as glycolysis and lactate metabolism, is gaining traction as a complementary therapeutic approach^[59,60]^.

## Targeting NETs and their role in metastasis

NETs have recently been implicated in breast cancer metastasis by facilitating CTC survival and extravasation. NETs are web-like structures composed of DNA, histones, and proteases that can trap tumor cells and promote their adhesion to distant organs. Targeting NETs with DNase I, which degrades extracellular DNA, has been explored as a strategy to prevent metastasis in breast cancer. Additionally, inhibitors of peptidyl arginine deiminase 4, an enzyme critical for NET formation, have shown promise in reducing the metastatic burden in preclinical studies.

## Targeting tumor-derived exosomes and their role in immune modulation

Exosomes are small extracellular vesicles secreted by tumor cells that facilitate intercellular communication within the TME. They play a significant role in immune modulation, drug resistance, and metastasis by transferring oncogenic factors to recipient cells. Targeting tumor-derived exosomes using exosome inhibitors, such as GW4869 and dimethyl amiloride, has been explored as a strategy to disrupt tumor-stroma interactions and enhance the efficacy of immunotherapies. Additionally, leveraging engineered exosomes for targeted drug delivery presents an innovative approach to overcoming therapy resistance in breast cancer^[61]^.

## Advancing clinical applications in breast cancer

Breast cancer remains one of the leading causes of cancer-related morbidity and mortality worldwide. Despite advancements in early detection and treatment, challenges such as drug resistance, tumor recurrence, and metastasis continue to hinder the effectiveness of existing therapies. Over the years, clinical applications have evolved to encompass novel therapeutic approaches, diagnostic innovations, and personalized medicine strategies, offering new hope for improved patient outcomes. The integration of targeted therapies, immunotherapies, advanced imaging techniques, and liquid biopsy has revolutionized the clinical landscape of breast cancer management. These innovations not only enhance early detection but also improve treatment precision, leading to better prognostic outcomes and reduced side effects^[62]^.

## Targeted therapy and precision medicine in breast cancer

The advent of targeted therapy has transformed breast cancer treatment, allowing for interventions that specifically address the molecular and genetic characteristics of tumors. Unlike traditional chemotherapy, which affects both cancerous and healthy cells, targeted therapies minimize damage to normal tissues and reduce systemic toxicity. One of the most significant breakthroughs in targeted therapy has been the development of human epidermal growth factor receptor 2 (HER2)-targeted agents. HER2-positive breast cancer accounts for approximately 15–20% of all breast cancer cases and is characterized by the overexpression of the HER2, which drives aggressive tumor growth. The introduction of trastuzumab (Herceptin), a monoclonal antibody that binds to the HER2 receptor and inhibits its signaling, has dramatically improved survival rates for HER2-positive patients. Following trastuzumab, newer HER2-targeted drugs such as pertuzumab, ado-trastuzumab emtansine (T-DM1), and trastuzumab deruxtecan have been developed, further enhancing treatment efficacy and addressing resistance mechanisms. Similarly, hormone receptor-positive (HR+) breast cancer, which constitutes the majority of breast cancer cases, has benefited from targeted endocrine therapies. Drugs such as tamoxifen and aromatase inhibitors (letrozole, anastrozole, and exemestane) have been widely used to block estrogen signaling, thereby inhibiting tumor growth. The introduction of cyclin-dependent kinase 4/6 inhibitors – palbociclib, ribociclib, and abemaciclib – has further improved outcomes for HR+ breast cancer patients by disrupting cell cycle progression and preventing uncontrolled tumor proliferation. In triple-negative breast cancer (TNBC), which lacks HER2, estrogen, and progesterone receptors, targeted therapy has been more challenging due to the absence of specific molecular targets. However, recent advances in PARP (poly ADP-ribose polymerase) inhibitors such as olaparib and talazoparib have provided new therapeutic avenues for patients with BRCA1/2 mutations, exploiting defects in DNA repair pathways to selectively kill cancer cells^[63-65]^.

## Immunotherapy and checkpoint inhibitors

The role of immunotherapy in breast cancer has gained significant attention, particularly with the approval of immune checkpoint inhibitors for specific breast cancer subtypes. Unlike conventional treatments that directly target cancer cells, immunotherapy leverages the body’s immune system to recognize and destroy malignant cells. One of the most notable advancements has been the use of programmed cell death 1 (PD-1)/programmed death-ligand 1 (PD-L1) checkpoint inhibitors, such as pembrolizumab and atezolizumab, in the treatment of TNBC. TNBC is known for its aggressive nature and limited treatment options; however, checkpoint blockade therapy has demonstrated significant benefits in patients with PD-L1-positive tumors by reinvigorating exhausted T cells and restoring anti-tumor immune responses. The combination of checkpoint inhibitors with chemotherapy has further improved survival rates, marking a major milestone in breast cancer immunotherapy. Another emerging immunotherapeutic strategy involves the development of cancer vaccines targeting specific tumor antigens. While still in clinical trials, vaccines such as NeuVax, which targets HER2-expressing tumors, and vaccines against mammaglobin-A, a breast-specific antigen, hold promise in preventing recurrence and boosting long-term immunity^[66,67]^.

## Liquid biopsy and early detection strategies

Early and accurate detection of breast cancer is crucial for improving survival rates, and liquid biopsy has emerged as a revolutionary non-invasive diagnostic tool. Unlike traditional tissue biopsies, which require surgical intervention, liquid biopsy enables real-time monitoring of tumor evolution through the detection of circulating tumor DNA (ctDNA), CTCs, and exosomal biomarkers in the blood.

Clinical applications of liquid biopsy include:

- **Early Diagnosis**: Liquid biopsy can detect cancer-related genetic alterations even before tumors become detectable through conventional imaging techniques. This allows for early intervention and improved treatment outcomes.
- **Monitoring Treatment Response**: By analyzing ctDNA levels, clinicians can assess how well a patient is responding to therapy and make necessary adjustments to treatment plans.
- **Identifying Minimal Residual Disease (MRD)**: Liquid biopsy can detect microscopic traces of cancer that remain after treatment, helping to identify patients at risk of relapse and guiding decisions on additional therapy.
- **Detecting Resistance Mutations**: Tumors can develop resistance to targeted therapies over time. Liquid biopsy enables the detection of resistance mutations, such as ESR1 mutations in hormone therapy-resistant breast cancer or HER2 mutations in trastuzumab-resistant cases, allowing for timely modifications in treatment strategy^[68,69]^.

## Artificial intelligence and advanced imaging in breast cancer diagnosis

The integration of artificial intelligence (AI) and machine learning in breast cancer diagnostics has significantly improved early detection and risk assessment. AI-powered imaging tools can analyze mammograms, ultrasounds, and magnetic resonance imaging (MRI) scans with high precision, reducing false positives and improving diagnostic accuracy.

- **AI-Enhanced Mammography**: AI algorithms have been developed to detect subtle patterns in mammograms that may be missed by human radiologists. These tools can help prioritize high-risk cases and facilitate early-stage diagnosis.
- **Radiomics and Personalized Imaging**: Advanced imaging techniques, such as radiomics, extract quantitative features from imaging data to predict tumor aggressiveness and response to treatment. This information aids in tailoring personalized treatment plans.
- **AI-Driven Pathology**: Digital pathology platforms utilize AI to analyze histological slides, identifying key biomarkers and predicting patient prognosis with greater accuracy^[70,71]^.

## Metabolic targeting and novel therapeutics

Cancer cells exhibit altered metabolism to sustain rapid growth and survival. Targeting metabolic pathways has gained traction as a promising therapeutic approach in breast cancer.

- **Inhibiting Glycolysis**: Breast cancer cells often rely on glycolysis (Warburg effect) to generate energy, even in the presence of oxygen. Glycolytic inhibitors such as 2-deoxy-D-glucose and pyruvate kinase M2 inhibitors are being explored as potential breast cancer treatments.
- **Targeting Glutamine Metabolism**: Certain breast cancer subtypes, such as TNBC, are highly dependent on glutamine metabolism. Inhibitors of glutaminase, such as CB-839, are being investigated in clinical trials to disrupt this metabolic dependency.
- **Exploiting Lipid Metabolism**: Lipid biosynthesis is crucial for cancer cell proliferation. Inhibitors of fatty acid synthase and lipid-lowering drugs such as statins are being studied for their potential anti-tumor effects in breast cancer^[72,73]^.

## Mechanistic insights into breast cancer

Breast cancer is a highly heterogeneous malignancy with a complex molecular landscape that dictates its behavior, response to treatment, and metastatic potential. At the heart of this disease lies a network of interwoven signaling pathways, epigenetic modifications, immune interactions, and metabolic reprogramming that collectively drive tumor initiation, progression, and therapy resistance.

## The hormone receptor axis: orchestrating tumor growth and survival

A large proportion of breast cancers rely on hormone receptor signaling for growth, particularly those classified as estrogen receptor-positive (ER+). Estrogen, a key driver in these cancers, exerts its oncogenic effects through both genomic and non-genomic pathways. In the classical genomic pathway, estrogen binds to the estrogen receptor (ER), inducing conformational changes that facilitate receptor dimerization and nuclear translocation. Once in the nucleus, the ER complex binds to estrogen response elements (EREs) on DNA, recruiting coactivators such as steroid receptor coactivator-3 and CREB-binding protein. This cascade ultimately activates transcription of genes involved in cell cycle progression, such as cyclin D1, which drives the transition from G1 to S phase, and BCL2, which promotes cell survival by inhibiting apoptosis. Beyond direct transcriptional regulation, ER also engages in cross-talk with growth factor receptors, particularly HER2 and IGF-1R, through membrane-associated signaling. This non-genomic mechanism enables rapid activation of intracellular kinases such as phosphoinositide 3-kinase (PI3K)/Akt and mitogen-activated protein kinase (MAPK), reinforcing a pro-survival and anti-apoptotic state. These parallel pathways contribute to endocrine resistance, a major therapeutic challenge in ER+ breast cancer. The use of selective ER degraders and inhibitors targeting downstream kinases such as PI3K has emerged as an effective strategy to counteract resistance^[74,75]^.

## HER2-driven oncogenesis: amplifying malignant signaling

HER2-positive breast cancers, which account for approximately 15–20% of cases, are characterized by overexpression or amplification of the HER2 gene. Unlike other members of the HER family, HER2 lacks a direct ligand but is constitutively active upon dimerization with HER1, HER3, or HER4. This persistent activation results in uncontrolled downstream signaling through key oncogenic pathways. The PI3K/Akt pathway is particularly critical in HER2-driven tumors. Upon HER2 activation, PI3K phosphorylates and activates Akt, which in turn inhibits pro-apoptotic proteins such as BAD and enhances the activity of mTORC1, a master regulator of protein synthesis and cellular metabolism. The net result is an increase in cellular proliferation, survival, and metabolic adaptability. Concomitantly, HER2 activation triggers the RAS/RAF/MAPK pathway, promoting transcription of genes involved in cell cycle progression and epithelial–mesenchymal transition (EMT). The latter process is crucial for metastasis, as it enables epithelial cells to lose their adhesive properties and gain migratory capabilities. Tumor cells undergoing EMT exhibit downregulation of E-cadherin and upregulation of mesenchymal markers such as vimentin and N-cadherin, facilitating invasion into surrounding tissues and dissemination to distant organs. Targeting HER2 has been a major breakthrough in breast cancer therapy. Monoclonal antibodies such as trastuzumab and pertuzumab block HER2 dimerization, whereas tyrosine kinase inhibitors like lapatinib and neratinib inhibit downstream signaling. Additionally, antibody–drug conjugates, such as T-DM1, deliver cytotoxic agents specifically to HER2-expressing cells, minimizing systemic toxicity while maximizing anti-tumor efficacy^[76,77]^.

## TNBC: the complexity of aggression and resistance

Unlike ER+ and HER2+ breast cancers, TNBC lacks hormone receptor and HER2 expression, making it particularly challenging to treat. TNBC is a highly aggressive subtype characterized by genetic instability, immune evasion, and metabolic adaptations that drive resistance to conventional therapies. One of the hallmarks of TNBC is the dysregulation of DNA damage response pathways. Many TNBC tumors harbor BRCA1/2 mutations, rendering them deficient in homologous recombination repair. This vulnerability has been therapeutically exploited using poly (ADP-ribose) polymerase (PARP) inhibitors such as olaparib and talazoparib, which induce synthetic lethality by preventing tumor cells from repairing DNA damage. TNBC also exhibits a profound ability to evade immune surveillance. Tumor cells upregulate PD-L1, which binds to PD-1 receptors on T cells, leading to immune exhaustion. Additionally, the TME of TNBC is often enriched with TAMs that secrete immunosuppressive cytokines such as IL-10 and TGF-β. These factors create an immunosuppressive niche, allowing TNBC cells to proliferate unchecked. Immune checkpoint inhibitors such as pembrolizumab and atezolizumab aim to reverse this immune evasion by reactivating T-cell responses against TNBC cells. Metabolic reprogramming further supports TNBC survival. Unlike hormone receptor-positive tumors that rely heavily on oxidative phosphorylation, TNBC cells preferentially engage in glycolysis, even in the presence of oxygen (the Warburg effect). This metabolic shift allows rapid ATP production and the generation of biosynthetic precursors necessary for proliferation. Targeting key metabolic enzymes such as hexokinase-2 and lactate dehydrogenase A is being explored as a therapeutic approach in TNBC^[78-80]^.

## The TME

Beyond intrinsic signaling networks, breast cancer progression is profoundly influenced by its microenvironment, composed of immune cells, fibroblasts, ECM components, and soluble factors.

- **CAFs**: These fibroblasts secrete ECM proteins and growth factors that facilitate tumor growth and invasion. CAF-derived TGF-β promotes EMT, while FAP enhances immunosuppression.
- **TAMs**: Macrophages within the TME can adopt an M2-like phenotype, secreting cytokines that suppress anti-tumor immunity and promote angiogenesis.
- **Neutrophils and N1/N2 Polarization**: Neutrophils exhibit plasticity in the TME, functioning either as anti-tumorigenic (N1) or pro-tumorigenic (N2) cells. The latter supports tumor progression by releasing pro-angiogenic factors and suppressing cytotoxic immune responses.
- **HIFs**: Hypoxic conditions in the TME activate HIF-1α, which upregulates VEGF, promoting angiogenesis and resistance to therapy^[79,80]^.

## Groundbreaking discoveries in breast cancer

Breast cancer research is at the forefront of a revolution, driven by groundbreaking discoveries that are reshaping our understanding of tumor biology, metastasis, treatment resistance, and therapeutic interventions. From advanced genomic insights to the emergence of novel targeted therapies and immunological breakthroughs, these findings are paving the way for more precise and effective treatment strategies. As the scientific community continues to unravel the complexities of breast cancer, the focus remains on translating these discoveries into clinical applications that can significantly improve patient outcomes.

## Rewriting the genomic landscape: the era of whole genome sequencing (WGS)

One of the most transformative advancements in breast cancer research is the application of WGS to decode the genetic architecture of tumors at an unprecedented level of detail. Traditionally, genetic profiling focused on a limited set of mutations within well-characterized oncogenes such as *TP53, PIK3CA, BRCA1*, and *HER2.* However, recent large-scale studies, including The Cancer Genome Atlas and the METABRIC project, have expanded the understanding of breast cancer heterogeneity by identifying novel driver mutations, structural variants, and regulatory elements that were previously overlooked. A striking discovery from WGS is the role of enhancer hijacking in aggressive breast cancer subtypes. Enhancer elements, which regulate gene expression, can be aberrantly activated due to structural rearrangements, leading to the overexpression of oncogenes. This mechanism has been implicated in TNBC, where the rearrangement of enhancer regions results in the upregulation of MYC, a master regulator of tumor proliferation and survival. These findings open new avenues for therapeutic interventions targeting enhancer activity through small-molecule inhibitors and CRISPR-based epigenetic editing^[81,82]^.

## Single-cell sequencing and tumor evolution

The advent of single-cell RNA sequencing has provided unparalleled insights into the cellular heterogeneity of breast tumors. Unlike bulk sequencing, which averages gene expression across a tumor mass, single-cell approaches reveal the dynamic interplay between different cell populations within the TME. Recent studies have uncovered the presence of distinct cancer cell subpopulations that contribute to therapy resistance and metastatic progression. For instance, researchers have identified a rare population of therapy-resistant cells characterized by a hybrid epithelial–mesenchymal (E/M) phenotype. These cells exhibit both epithelial adhesion properties and mesenchymal motility, allowing them to evade chemotherapy and migrate to distant organs. Targeting this population with drugs that disrupt the EMT process has emerged as a promising strategy to prevent metastasis and improve long-term patient survival. Furthermore, single-cell sequencing has revolutionized the understanding of immune evasion in breast cancer. Tumors often develop mechanisms to suppress immune surveillance, leading to resistance against immunotherapies such as checkpoint inhibitors. By profiling immune cell populations at the single-cell level, researchers have discovered novel subsets of exhausted T cells and immunosuppressive macrophages that contribute to immune evasion. This knowledge has paved the way for combination therapies that reinvigorate anti-tumor immunity, such as the use of anti-PD-1 inhibitors alongside immune-modulating agents like IL-12 or STING agonists^[83]^.

## Targeting dormant tumor cells

A longstanding challenge in breast cancer treatment is the phenomenon of tumor dormancy, where cancer cells remain in a quiescent state for years or even decades before reactivating to form metastatic lesions. These dormant cells evade conventional chemotherapy, which primarily targets actively dividing cells, leading to late-stage recurrences that are often untreatable. Recent breakthroughs have identified key molecular regulators of tumor dormancy, shedding light on potential therapeutic targets. One of the most compelling findings is the role of the NR2F1 transcription factor in maintaining dormancy. High levels of NR2F1 suppress proliferative signals and promote a quiescent state, while its downregulation triggers reactivation and metastatic outgrowth. Pharmacological activation of NR2F1 using small molecules has shown promising results in preclinical models, preventing the awakening of dormant cells and reducing metastatic burden. Additionally, emerging research has linked tumor dormancy to metabolic adaptations. Dormant breast cancer cells exhibit a distinct metabolic profile, relying on oxidative phosphorylation rather than glycolysis for energy production. This dependency on mitochondrial metabolism presents a unique vulnerability that can be exploited through drugs targeting oxidative phosphorylation pathways, such as mitochondrial inhibitors or AMPK activators^[84,85]^.

## Synthetic lethality in breast cancer

The concept of synthetic lethality – where targeting two pathways simultaneously leads to tumor cell death while sparing normal cells – has emerged as a groundbreaking approach in breast cancer therapy. This strategy has already been successfully implemented in BRCA-mutated tumors using PARP inhibitors, which exploit defects in DNA repair to induce synthetic lethality. Beyond BRCA mutations, researchers have identified novel synthetic lethal interactions that could expand therapeutic options for a broader range of breast cancer patients. One such discovery involves the dependency of PIK3CA-mutant tumors on the enzyme ATAD5, which regulates DNA replication stress. Inhibition of ATAD5 in PIK3CA-mutant cells leads to catastrophic DNA damage and tumor cell death, highlighting a promising new target for drug development. Moreover, synthetic lethality has been leveraged to overcome resistance to endocrine therapy in ER-positive breast cancer. A recent study revealed that blocking CDK7, a key regulator of transcriptional elongation, selectively induces apoptosis in endocrine-resistant tumors while sparing normal breast cells. This finding has led to the development of CDK7 inhibitors, which are currently being evaluated in clinical trials as a potential solution to treatment resistance^[86,87]^.

## Liquid biopsies: revolutionizing early detection and monitoring

The rise of liquid biopsy technology has transformed the landscape of breast cancer diagnostics, offering a minimally invasive method to detect and monitor cancer in real time. Traditional tissue biopsies provide a static snapshot of the tumor at a single time point, whereas liquid biopsies enable dynamic tracking of tumor evolution through the analysis of ctDNA, CTCs, and extracellular vesicles. Recent breakthroughs have demonstrated that ctDNA analysis can detect MRD months or an even year before clinical relapse occurs. This early detection capability allows for the initiation of treatment before overt metastasis develops, significantly improving patient outcomes. Additionally, liquid biopsies have proven invaluable in predicting treatment response, enabling personalized adjustments to therapy based on real-time molecular profiling. The application of liquid biopsies extends beyond early detection to the realm of treatment resistance. By sequencing ctDNA over time, researchers have identified novel resistance mechanisms to targeted therapies, such as secondary mutations in *ESR1* that drive resistance to endocrine therapy. These insights are now guiding the development of next-generation inhibitors designed to overcome acquired resistance and extend treatment efficacy^[88,89]^.

## Neutrophil polarization in cancer: a spectrum of phenotypes

Neutrophils, the most abundant white blood cells in the body, have long been viewed as critical players in the innate immune response, primarily tasked with defending against infections. Traditionally, these cells have been classified based on their functional roles into two distinct polarization states: **N1** (anti-tumor) and **N2** (pro-tumor). However, recent research has revealed that neutrophil polarization is far more complex than this binary classification, resembling a dynamic spectrum rather than rigid phenotypes. This evolving understanding has important implications for their role in cancer, particularly in the TME, where neutrophils interact with a variety of other immune cells, tumor cells, and stromal components.

## The spectrum of neutrophil polarization

The idea of neutrophil polarization as a spectrum challenges the traditional binary model, suggesting that neutrophils do not strictly exist as either “N1” or “N2” but rather exhibit varying degrees of pro-inflammatory or anti-inflammatory behaviors based on the local environment and stimuli within the TME. This spectrum encompasses a range of intermediate states that can be influenced by multiple factors, including cytokines, chemokines, and metabolic cues. Importantly, neutrophil polarization is not fixed and can be influenced by both intrinsic and extrinsic signals, making these cells highly adaptable in response to the ever-changing TME^[48]^.

## Factors influencing neutrophil polarization

Neutrophils exhibit remarkable plasticity, with polarization driven by a variety of external factors within the TME. The primary elements that influence neutrophil polarization include:

1. **Cytokines and Chemokines**: The TME is rich in diverse cytokines and chemokines, such as **IL-6, IL-8, G-CSF, TGF-β**, and **TNF-α**, which play a critical role in shaping neutrophil behavior. For example, **IL-12** and **TNF-α** are associated with the promotion of the N1 phenotype, which is linked to pro-inflammatory responses, while **IL-4, IL-13**, and **TGF-β** are associated with N2 polarization, which tends to promote tissue repair, immune suppression, and tumor progression.
2. **Hypoxia**: A hallmark of solid tumors is hypoxia, which can influence the polarization of neutrophils in the TME. HIFs, particularly HIF-1α, play a key role in directing neutrophil behavior, often skewing them toward the N2 phenotype, which promotes angiogenesis, tissue remodeling, and immune suppression.
3. **Metabolic Changes**: Tumors are metabolically active environments, and the altered metabolism within tumors, such as increased glycolysis, can influence neutrophil function. Metabolic shifts in neutrophils, including changes in their glycolytic and oxidative metabolism, can lead to polarization toward different phenotypes, depending on the tumor’s needs.
4. **Tumor-Derived Factors**: Tumor cells themselves secrete a variety of factors that can push neutrophils toward different polarization states. For instance, **VEGF**, produced by tumor cells, is a key factor in promoting the N2 polarization of neutrophils by enhancing angiogenesis and promoting an immunosuppressive environment. In contrast, cytokines such as **IL-17** and **GM-CSF** can encourage a more pro-inflammatory phenotype, possibly leaning toward N1-like activity^[48,89]^.

## The intermediate phenotypes: beyond N1 and N2

While the N1 and N2 dichotomy provides useful shorthand for understanding the roles of neutrophils in cancer, there is growing recognition that neutrophils can exhibit characteristics of both phenotypes or exist in more transitional or intermediate states. These intermediate states are often shaped by the dynamic and fluid nature of the TME, where neutrophils constantly respond to changing signals.

1. **Plasticity of Neutrophils**: Neutrophils are capable of shifting between pro-inflammatory (N1-like) and pro-tumor (N2-like) states in response to cues from the TME. For instance, neutrophils that initially adopt an N1-like phenotype in response to anti-tumor cytokines may switch to an N2-like phenotype if exposed to factors such as TGF-β or hypoxia. This plasticity reflects the ability of neutrophils to not only adapt but also potentially exacerbate disease progression under certain conditions, such as metastasis and therapy resistance.
2. **Transitional States and Dual Functionality**: Research has also shown that neutrophils within the TME can exhibit dual functionality, displaying a hybrid phenotype that incorporates characteristics of both N1 and N2 neutrophils. For example, these cells may simultaneously produce pro-inflammatory cytokines while also promoting tumor cell survival and metastasis through the secretion of angiogenic factors. Such transitional states complicate the therapeutic targeting of neutrophils, as their functions may shift over time or in response to treatment.
3. **Regulation of Tumor Progression**: The intermediate polarization states of neutrophils in the TME can influence tumor progression in multiple ways. On one hand, neutrophils with a hybrid phenotype may contribute to tissue remodeling, creating a more favorable environment for tumor cells to invade and spread. On the other hand, they may also facilitate immune evasion, suppressing effective anti-tumor responses through the recruitment of immunosuppressive cells, such as regulatory T cells (Tregs), or by producing immunosuppressive cytokines like IL-10 and TGF-β^[88,89]^.

## The role of neutrophils in tumor growth and metastasis

The spectrum of neutrophil polarization plays a significant role in tumor biology, particularly in the processes of tumor growth, metastasis, and immune evasion.

1. **Tumor Growth**: N1 neutrophils are typically associated with inhibiting tumor growth through cytotoxic mechanisms and the enhancement of immune responses. However, intermediate and N2-like neutrophils can promote tumor cell proliferation and survival. These neutrophils create a favorable microenvironment by promoting angiogenesis, ECM remodeling, and immune suppression.
2. **Metastasis**: Neutrophils, particularly those with N2-like features, contribute to metastasis by facilitating tumor cell invasion and dissemination. They do so by secreting MMPs, which degrade the ECM, and by fostering an immunosuppressive environment that allows tumor cells to evade immune surveillance. This process is further enhanced by the secretion of pro-angiogenic factors that facilitate the formation of new blood vessels, supporting the growth of metastatic lesions.
3. **Immune Evasion and Therapy Resistance**: As part of the TME, neutrophils can help tumor cells evade immune detection, particularly through the N2 polarization. These neutrophils may suppress the activity of cytotoxic T cells and NK cells, both of which are essential for anti-tumor immunity. Moreover, neutrophils with an N2 phenotype are often implicated in resistance to therapies, including chemotherapy and immunotherapy, by altering the TME and promoting tumor cell survival^[13,90]^.

## Therapeutic implications: targeting neutrophil polarization

Given the plasticity and spectrum of neutrophil polarization, targeting neutrophil function presents an exciting avenue for cancer therapy. Current strategies to modulate neutrophil polarization include:

1. **Cytokine Modulation**: Therapies aimed at manipulating key cytokines like IL-12, TNF-α, TGF-β, and IL-4 may be used to push neutrophils toward an N1-like phenotype, enhancing anti-tumor immunity. Conversely, blocking pro-tumor cytokines may inhibit the transition to the N2 phenotype and reduce tumor progression.
2. **Chemokine Receptor Blockade**: Targeting specific chemokine receptors, such as CXCR1 and CXCR2, may help prevent neutrophil recruitment to the TME, thereby reducing their potential to support tumor growth and metastasis.
3. **Neutrophil Reprogramming**: Recent studies have explored the possibility of reprogramming neutrophils to adopt an N1-like phenotype using small molecules or gene therapy approaches. This strategy holds promise in enhancing the anti-tumor immune response and improving the efficacy of existing therapies^[91]^.

## Therapeutic strategies targeting neutrophil polarization

Therapeutic strategies targeting neutrophil polarization hold significant promise in modulating the immune response in breast cancer, particularly for manipulating the balance between N1 and N2 neutrophils to favor antitumor immunity and inhibit metastasis. The goal of these therapies is to either enhance the tumor-suppressive functions of N1 neutrophils or reprogram N2 neutrophils to adopt a more antitumor phenotype. These approaches aim to reshape the TME, limit cancer progression, and improve the efficacy of existing cancer treatments, such as chemotherapy and immunotherapy^[53]^. One promising strategy to enhance N1 neutrophil function is the use of cytokines or small molecules that activate neutrophils and promote their polarization toward the N1 phenotype. For instance, granulocyte G-CSF and IFNs, especially IFN-γ, have been shown to induce the polarization of neutrophils into an N1 phenotype. These cytokines not only enhance neutrophil recruitment to the TME but also stimulate their cytotoxic activity, including the production of ROS, NETs, and pro-inflammatory cytokines that help suppress tumor growth. Additionally, the use of TLR (Toll-like receptor) agonists can also drive neutrophils toward an N1-like phenotype, improving their antitumor activity and boosting their ability to recruit other immune cells like CTLs and NK cells, which contribute to tumor cell destruction^[92]^.

Conversely, a key therapeutic approach targeting N2 neutrophils involves the inhibition of signaling pathways that drive their polarization or the use of agents that can reprogram N2 neutrophils to adopt a more antitumor phenotype. Several factors contribute to the skewing of neutrophils toward the N2 phenotype in the TME, including the presence of cytokines such as interleukin-4 (IL-4), interleukin-13 (IL-13), and TGF-β. These cytokines activate pathways such as the STAT6 and SMAD signaling pathways, which promote N2 polarization. Targeting these pathways with inhibitors, monoclonal antibodies, or small molecule inhibitors can disrupt N2 polarization, potentially reversing the pro-tumor effects of N2 neutrophils. In addition to inhibiting the TGF-β signaling pathway, targeting the chemokine receptor CCR2, which is involved in the recruitment of N2 neutrophils, may prevent their accumulation in the TME, reducing their tumor-promoting effects and enhancing the effectiveness of other treatments^[93,94]^.

Another approach involves utilizing immune checkpoint inhibitors, which are widely used in cancer immunotherapy, to enhance the antitumor function of neutrophils. Immune checkpoint molecules such as PD-1 and PD-L1 are known to contribute to immune suppression by promoting the accumulation of immunosuppressive cells, including N2 neutrophils, within the TME. By inhibiting the PD-1/PD-L1 axis, it is possible to reverse the immunosuppressive effects of N2 neutrophils and encourage a shift toward an N1 phenotype. This strategy has shown promise in preclinical models and is being investigated in combination with other therapies to enhance the overall immune response against breast cancer. Moreover, the combination of checkpoint inhibitors with chemotherapy or radiation therapy can enhance the recruitment of N1 neutrophils to the tumor site, leading to improved tumor control and reduced metastasis^[58]^. Another emerging strategy involves the use of nanomedicine and drug delivery systems to specifically target and reprogram neutrophils within the TME. Nanoparticles or liposomes can be designed to carry therapeutic agents directly to the tumor site, minimizing off-target effects and enhancing the precision of the treatment. For example, nanoparticles loaded with anti-inflammatory cytokines or small molecules that inhibit the pro-tumor functions of N2 neutrophils can be used to promote neutrophil polarization toward the N1 phenotype. Additionally, the use of nanoparticles that can deliver specific gene-editing tools such as CRISPR/Cas9 could allow for the precise manipulation of neutrophil gene expression, further enabling the targeted reprogramming of neutrophil function in the TME^[95-101]^.

## Conclusion

Neutrophils play a critical role in the TME of breast cancer, with their polarization into N1 and N2 phenotypes influencing both tumor progression and metastasis. While N1 neutrophils exhibit antitumor activity by promoting immune responses and attacking cancer cells, N2 neutrophils often contribute to immune suppression, tumor growth, and metastasis. Emerging strategies aimed at modulating neutrophil polarization offer promising avenues for improving breast cancer treatment outcomes. By promoting the polarization of neutrophils toward the N1 phenotype or reprogramming N2 neutrophils to a more antitumor state, it may be possible to enhance the efficacy of immunotherapies, chemotherapy, and other targeted treatments. Despite the therapeutic potential, challenges remain in fully understanding the complexities of neutrophil biology, including their dual roles in cancer progression and the need for precise targeting to avoid unintended side effects.

## Data Availability

Not applicable as this a review.

## Research registration unique identifying number (UIN)

Not applicable as this a review.
